# Social and Poor-Law Problems

**Published:** 1908-07-18

**Authors:** 


### SOCIAL AND POOR-LAW PROBLEMS.

### ACCIDENT PATIENTS IN POOR-LAW INFIRMARIES.

The great improvements which have taken place in the equipment and management of Poor-law infirmaries of late years have led to their being used very largely as general hospitals; indeed, in some places there are no hospitals conveniently available, and even in London, when the voluntary hospitals are overcrowded, patients who are far from being paupers in any proper meaning of the term are sent to the infirmary. This is, of course, peculiarly common in the case of accidents when hospital treatment is necessary and there may be no bed vacant in the voluntary hospital of the vicinity. At the recent meeting of the Association of Poor-law Unions, the Toxteth Park Board of Guardians brought forward a resolution proposing that "persons admitted to workhouse hospitals through failure to obtain admission to other hospitals, and who contribute to the cost of their maintenance therein such amounts as are fixed by the Guardians, should not be classified as paupers, and should be exempt from disfranchisement." After consideration, however, the Council of the Association resolved to take no action, as to adopt the resolution would be to extend still further the provisions of the Medical Relief Disqualification Act. This may seem a hard decision, but Poor-law guardians are, as a rule, only too well aware of the danger of making dependence on the rates pleasant. If it were not for the dislike entertained to the word "pauper," there are many who would not scruple to take advantage of the workhouse infirmary—especially when, as in many large unions, it is a building entirely distinct from and not inevitably even near the workhouse. The meanness of those who, not being necessitous, take advantage of the accommodation and treatment at voluntary hospitals is quite bad enough, but voluntary hospitals are maintained by the freewill contributions of those who feel that they can spare something for their sick fellows. But the Poor-law institutions are maintained by compulsory contributions from people, many of whom—indeed, the majority—find it difficult enough to make ends meet; their money should be taken only for the needs of the genuinely poor. It may be that in exceptional circumstances others than these may have occasionally to be received in the infirmaries; but if once the disqualifications attending residence in these were removed, the demands on the accommodation would be such as it would be almost impossible to cope with. It would not be accidents only which would seek to be admitted. As it is, there are too many people in the receipt of sufficient wages who are willing to send their sick to the infirmary, and the payment which guardians insist on forms but a slight protection against imposition, partly because even when promised it is not always faithfully paid and partly because there are people quite well enough off to pay what is demanded and quite callous enough to be glad of sick relatives at the price. A case recently came before the Lambeth Guardians, in which a woman was received into the infirmary although her husband was in receipt of £15 a week. The only check on such people is the disgrace which still attaches to the word "pauper," and if that were withdrawn, the need of increasing infirmary accommodation would add a serious additional amount to a rate which most people find sufficiently heavy. The real remedy is either an addition to the number and size of voluntary hospitals or a more rigid exclusion from the latter of cases which do not actually need hospital treatment.

